# Comprehensive Evaluation of Agronomic Traits and Nutritional Composition in Summer-Sown Vegetable Soybean Varieties from Shanghai, China

**DOI:** 10.3390/foods15132382

**Published:** 2026-07-03

**Authors:** Biting Cao, Lihua Zhu, Jiaqi You, Yuan Yuan, Weihong Gu, Hongjuan Yang, Duo Lv, Qingzhu Li, Chaohan Li

**Affiliations:** 1 Shanghai Key Laboratory of Protected Horticultural Technology, Protected Horticultural Research Institute, Shanghai Academy of Agricultural Sciences, Shanghai 201403, China; caobis@163.com (B.C.); zhulihua.007@163.com (L.Z.); youjiaqi270@sina.com (J.Y.); yuan_23@saas.sh.cn (Y.Y.); guwh518@163.com (W.G.); yanghj681114@163.com (H.Y.); 18298345890@163.com (D.L.); 2 Forest and Pomology Research Institute, Shanghai Academy of Agricultural Sciences, Shanghai 201403, China; 1984yu1986@163.com

**Keywords:** vegetable soybean, landrace, germplasm evaluation, nutritional quality, isoflavones, saponins

## Abstract

Shanghai-native vegetable soybean (*Glycine max* [L.] Merril) landraces are valuable germplasms, but their systematic evaluation for agronomic and nutritional traits remains insufficient. This study aimed to assess their phenotypic and nutritional diversity to explore their potential for breeding and meeting dietary needs. Twenty-nine local landraces and one control cultivar (‘Qingsu 7’) were evaluated for key agronomic traits, yield components, nutritional traits, and isoflavone profiles, using hierarchical clustering, principal component analysis (PCA), and correlation analysis. Substantial phenotypic diversity was found, with the germplasm classified into four groups. First pod height and effective pods per plant were highly variable. Nutritional traits showed low variability for crude protein but high diversity for crude fat, soluble sugars (dominated by sucrose), vitamin C, and free amino acids. Total isoflavone content in dry seeds varied widely, with genistin, daidzin, and daidzein as the main forms. ‘Xiangshui Maodou’ had high free amino acids and vitamin C, ‘Heiyan Susudou’ showed superior soluble sugar content, and two landraces exceeded 1500 μg/g DW total isoflavones. The landraces possess rich phenotypic diversity and nutritional diversity. This germplasm represents a valuable resource for breeding programs to enhance crop quality and address global nutritional demands.

## 1. Introduction

Vegetable soybean (*Glycine max* [L.] Merril), also called ‘edamame’, refers to soybeans harvested at the immature green stage, when the seeds have expanded to 80–90% of the pod width and display bright green coloration in both pods and seeds [1]. This type of soybean forms a distinct group separate from grain soybean [2]. Compared to grain soybean, vegetable soybean has larger pods and seeds, along with a sweeter taste, smoother texture, and more desirable flavor [3,4].

With a cultivation history of over 2000 years in China, the country currently contributes to more than 90% of global vegetable soybean production [2,5]. The optimal harvest time occurs approximately 35–39 days after flowering, corresponding to the R6 growth stage [6]. Only the beans are consumed, typically from pods containing two or three seeds, which are considered marketable [7].

In recent years, global demand for vegetable soybean has risen steadily, driven by its appealing sensory properties and high nutritional value. Fresh edamame seeds contain 582 kcal of energy per 100 g, along with 11.4 g protein, 7.4 g carbohydrates, 6.6 g fat, 100 mg vitamin A, 0.27 mg vitamin B_1_, 0.14 mg vitamin B_2_, 1 mg vitamin B_3_, 27 mg vitamin C, 140 mg phosphorus, 70 mg calcium, 1.7 mg iron and 140 mg potassium [8]. They are also rich in dietary fiber, essential amino acids, and bioactive compounds such as isoflavones [3,9]. However, several anti-nutritional factors, including tannins, protease inhibitors and phytic acid, have also been detected in edamame seeds [8]. The edible quality of vegetable soybean is primarily determined by key sensory attributes such as sweetness, umami, texture, and flavor [10,11], all of which are influenced by biochemical components, such as soluble sugars (mainly sucrose), free amino acids, crude fat, and crude protein [12]. Specifically, sucrose serves as the primary contributor to sweetness, free amino acids enhance both sweetness and umami, and crude fat and protein significantly affect texture and mouthfeel [13]. Notably, sweetness and umami are critical factors influencing consumer preference [14,15], while certain components, such as isoflavones, may contribute to undesirable bitterness in some products [16]. In addition to sensory traits, agronomic performance is another critical determinant of commercial success, encompassing traits like plant height, yield, seed size, and maturity time. Nevertheless, these traits exhibit complex interrelationships [17], as illustrated by the negative correlation between protein content and sweetness [18], and the trade-off between superior eating quality and germination efficiency [17]. These interactions are further modulated by genetic and environmental factors, including cultivar type, growing conditions, and soil properties, all contributing to significant phenotypic variation [6].

This variation is particularly evident in Shanghai, China, where long-term cultivation has produced numerous local landraces such as ‘NiuTaBian’, ‘ZaoLvPi’, and ‘GuLiQing’, traditionally distinguished by harvest period or seed morphology. Some of these indigenous cultivars, such as ‘NiuTaBian’, have been used in breeding programs due to their favorable traits such as heat tolerance and large pod size [19]. Nevertheless, the distinction between grain-type and vegetable-type soybeans remains ambiguous in some traditional varieties, highlighting the need for systematic evaluation and utilization of local germplasm. Previous breeding efforts have largely prioritized yield, with limited focus on nutritional quality [20]. Enhancing phenotypic and nutritional diversity through germplasm collection and pre-breeding research is essential for developing high-yielding cultivars with improved nutritional profiles, including optimized levels of protein, fat, vitamins, and anti-nutritional factors.

To address these gaps, this study evaluates 29 Shanghai-native soybean landraces for morphological, agronomic, and nutritional traits. The specific objectives are to evaluate phenotypic and nutritional diversity among these local accessions and identify superior genotypes combining high yield with desirable quality attributes, including protein content, vitamin C, free amino acids, fatty acids, and isoflavone profiles in both fresh and dry seeds. The findings will provide valuable insights into the phenotypic diversity and nutritional diversity and potential economic value of these resources, supporting future breeding initiatives and strategic promotion of vegetable soybean in expanding markets.

## 2. Materials and Methods

### 2.1. Plant Materials

We selected 29 vegetable soybean germplasms from diverse geographical regions across Shanghai (Table S1) as test materials. The summer-cropping variety ‘Qingsu 7’, developed by our group, was used as a reference cultivar and benchmarked against the local landraces.

### 2.2. Field Trials and Trait Measurement

A total of 30 vegetable soybean germplasm resources were cultivated from 2023 to 2024 at the Huacao Experimental Station of the Shanghai Academy of Agricultural Sciences (121°19′ E, 31°13′ N). Official meteorological records show that the annual precipitation was 1226.1 mm in 2023 and 1475.0 mm in 2024. The plum rain period was 24 days in 2023 and 15 days in 2024. Frequent rainstorms and poor sunshine were observed in April of 2024. The temperature conditions were stable during soybean growth in both years. The above climatic variations only caused small fluctuations in soluble sugars, crude protein and isoflavone contents. No significant inter-annual divergence was detected for agronomic and nutritional traits. The experiment was arranged in a randomized complete block design with three replications. Each accession was planted in a 3 m × 3.5 m plot, with row spacing of 45 cm and plant spacing of 30 cm. Each accession was evaluated at the immature seed stage (R6–R7) when seeds had expanded to fill 80% to 90% of the pod width. All harvested materials were immediately placed into plastic bags and transported to the laboratory for subsequent analysis.

The vegetable soybean agronomic and yield traits measured in this study included growth period, hypocotyl color, pubescence color, corolla color, seed shape, seed coat color, hilum color, cotyledon color, plant height, stem diameter, number of nodes on main stem, number of branches, first pod height, number of effective pods per plant, length of standard pod, width of standard pod, 100-seed fresh weight (SFW), and 100-seed dry weight (SDW) [21,22]. Plant height was measured from the soil surface to the tip of the main stem. The number of pods per plant was recorded, and the fresh pod weight was determined for all pods harvested from every vegetable soybean. Additionally, 10 pods were randomly selected from each plant for pod length, width, and thickness [21]. SFW (g) was determined from freshly hulled seeds, while SDW (g) was determined from dried hulled seeds.

### 2.3. Analysis of Protein, Free Amino Acids, Crude Fat, and Isoflavones

Nutritional composition was also analyzed, focusing on concentrations of crude protein, crude fat, free amino acids, saponins, vitamin C, isoflavones, starch, and total sugar (sum of sucrose, glucose, and fructose) [23].

The crude protein content of beans was determined using the Kjeldahl nitrogen method [1]. First, 0.51 g of snippet was accurately weighed into a Kjeldahl flask, followed by the addition of 2 g of selenium mixture and 25 mL of concentrated H_2_SO_4_. The mixture was heated to boiling until the solution became clear greenish-green. After cooling, the digest was quantitatively transferred to a 100 mL volumetric flask, diluted to the mark with distilled water, and mixed thoroughly. An aliquot of 5 mL of the prepared solution was transferred to the distiller, and alkalized with approximately 5 mL of 30% NaOH. Distillation was carried out for approximately 10 min, using 10 mL of a 2% boric acid solution pre-mixed with indicator as the receiving solution. Then, rinsing the cooling tip with distilled water and titrating it with 0.01 N HCl solution made a blank determination.

The crude fat content in beans was quantified according to the hydrolysis-based method described by Hijrah et al. (2024) [1]. A total of 1–2 g of snippet was weighed into a beaker, followed by the addition of 30 mL of 25% HCL, 20 mL of distilled water, and several boiling chips. After hydrolysis, the mixture was filtered while hot, and the residue was washed with hot water until neutral. The filter paper containing the residue was dried in an oven at 100–105 °C, then placed in a filter paper wrapper and extracted with hexane at approximately 80 °C for 2–3 h. The hexane extract was then distilled, and the remaining residue (crude fat) was dried to constant weight and weighed.

Total free amino acid content in fresh beans was quantified using the ninhydrin chromogenic method [24]. A total of 1–2 g of snippet was extracted with 20 mL of 70% ethanol at 50 °C for 30 min under ultrasonication. After centrifugation, the supernatant was collected, and the residue was extracted again. The supernatants were combined and diluted to 50 mL with 70% ethanol. An aliquot (1.0 mL) of the extract was mixed with ninhydrin reagent and acetate buffer (pH 5.4), heated in a boiling water bath for 15 min, and cooled. The absorbance was measured at 570 nm. Quantification was performed using an external standard curve with leucine as the standard, and results were expressed as mg/g dry weight.

Soluble sugar and starch contents were determined by the anthrone sulfuric acid colorimetric method [25]. A total of 0.5 g of snippet was extracted with 80% ethanol at 80 °C for 30 min. After centrifugation, 1.0 mL of the supernatant was mixed with 4.0 mL of anthrone reagent and heated in a boiling water bath for 10 min, and the absorbance was measured at 620 nm. The soluble sugar content was calculated using a standard curve prepared with glucose. The residue was further extracted with 52% perchloric acid in an ice bath for 15 min. After centrifugation, 1.0 mL of the supernatant was collected and measured at 620 nm. The starch content was determined using a glucose calibration curve.

Vitamin C content in beans was estimated using the 2,6-dichloroindophenol dye method [25]. A portion of 10–40 g of fresh bean samples was homogenized with an equal mass of 2% oxalic acid solution, diluted to 100 mL, and filtered. An aliquot of 10 mL filtrate was titrated with standardized 2,6-dichloroindophenol solution until a pink color persisted for 15 s. A blank titration was performed using 2% oxalic acid solution, and the ascorbic acid content was calculated accordingly.

Saponins in beans were analyzed by high-performance liquid chromatography (HPLC) following the procedure of Berhow et al. (2000) [26]. A total of 2.0 g of snippet was extracted with 20 mL of 80% ethanol under ultrasonication for 30 min. After centrifugation, the supernatant was collected, and the residue was extracted once more under the same conditions. The supernatants were combined, diluted to 25 mL with 80% ethanol, and filtered through a 0.45 μm membrane prior to HPLC analysis. Chromatographic separation was performed on a C_18_ reversed-phase column (4.6 mm × 250 mm, 5 μm) at 30 °C. Gradient elution was carried out using acetonitrile as mobile phase A and 0.05% acetic acid aqueous solution as mobile phase B, at a flow rate of 1.0 mL/min. The detection wavelength was set at 205 nm. A standard curve was established using soybean saponin standards, and the saponin content was quantified using the external standard method based on peak area.

The isoflavone concentration was determined via HPLC (Agilent 1260 Infinity II, Waldbronn, Germany) according to Hoeck et al. (2000) [27]. A total of 2 g of beans was ground and placed in a 125 mL screw-capped flask, followed by the addition of 2 mL of 0.1 M HCL and 10 mL of acetonitrile (ACN). The mixture was stirred at room temperature for 2 h, after which 7 mL of distilled water was added. The solution was filtered through Whatman no. 42 filter paper, and the residue was rinsed three times with ACN. The filtrate was transferred to a 100 mL round-bottom flask and evaporated to dryness at 30 °C using a rotary evaporator. The dried residue was redissolved in 10 mL of 80% (*v*/*v*) methanol and transferred to a 10 mL volumetric flask. The redissolved material was filtered through a 0.45 μm filter, and 1 mL of the filtrate was transferred to a sample vial. A 20 μL aliquot of the filtrate was used for HPLC analysis. Seed preparation for HPLC analysis was performed as described by Kim et al. (2007) [28].

### 2.4. Statistical Analysis

All data were recorded and organized using Microsoft Excel (Version 2019). Data from the 2023 and 2024 growing seasons were first analyzed separately. As no significant differences were detected in agronomic traits and nutritional components between years, the datasets were combined for further analysis. Correlation analysis was performed using the bivariate method in SPSS (Version 27.01), with Pearson’s correlation coefficient used to assess relationships and two-tailed tests to determine statistical significance. Principal component analysis (PCA) was performed using SPSS (Version 27.01). Prior to analysis, data were standardized to eliminate dimensional differences. Principal component extraction was conducted based on the correlation matrix. Principal components with eigenvalues > 1 were retained according to the Kaiser criterion, and the number of retained principal components was determined by their cumulative variance contribution rate. Hierarchical cluster analysis was performed using SPSS (Version 27.01). After data standardization, Euclidean distance was used as the measurement index, and Ward’s minimum variance method was applied for clustering.

## 3. Results

### 3.1. Analysis of Vegetable Soybean Main Agronomic Traits and Yield Traits

**Figure 1:**
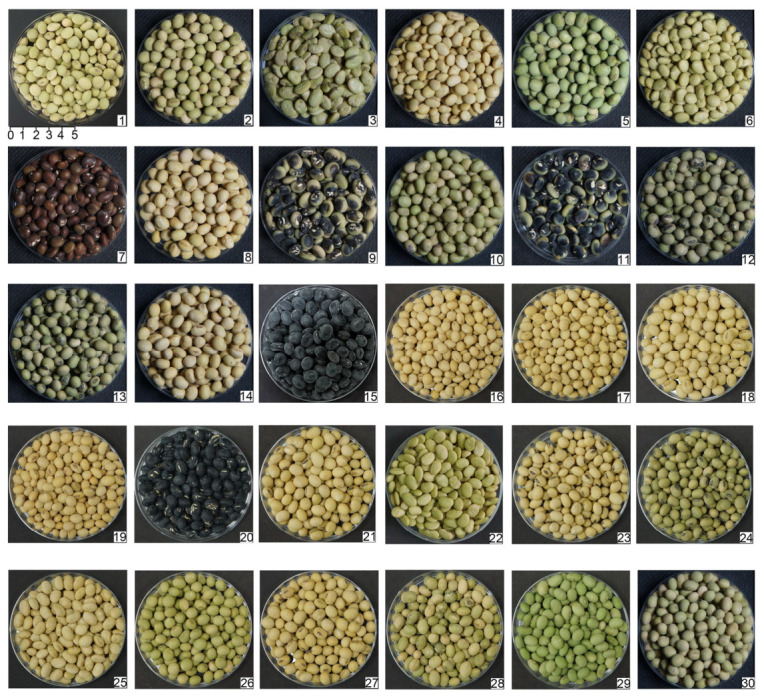
Morphological diversity of 30 vegetable soybean accessions.

**Figure 2:**
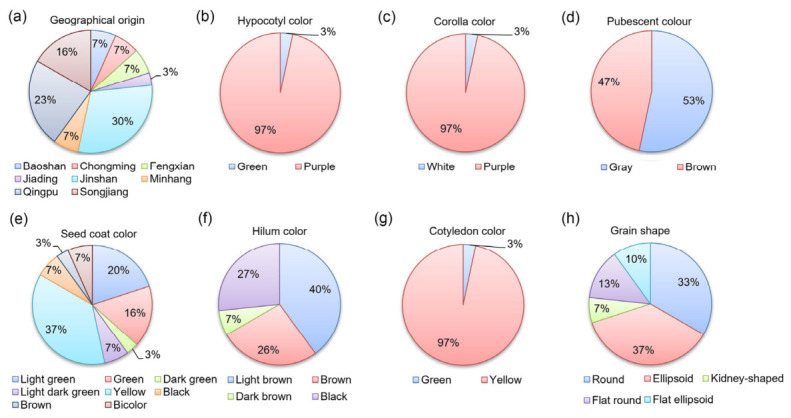
Characterization of morphological diversity in soybean germplasm. The pie charts depict the distribution of (**a**) geographical origin, (**b**) hypocotyl color, (**c**) corolla color, (**d**) pubescent color, (**e**) seed coat color, (**f**) hilum color, (**g**) cotyledon color, and (**h**) grain shape across the germplasm collection. Values represent the proportion of accessions exhibiting each trait variant. Numbers represent the proportion within the corresponding trait.

Significant variations in phenotypic traits were observed across the 30 vegetable soybean accessions (Figure 1, Table S1), collected from eight regions in Shanghai, primarily Qingpu District (Figure 2a). Most local accessions (29 out of 30) exhibited purple hypocotyls and corollas, while the control cultivar ‘Qingsu 7’ had green hypocotyls and white corollas (Table S1, Figure 2b,c). Among the 30 vegetable soybean accessions, 16 exhibited gray pubescence, while the remaining 14 had brown pubescence (Table S1, Figure 2d). The 30 vegetable soybean accessions exhibited a diverse range of seed coat colors, which comprised yellow (11), light green (6), green (5), dark green (1), light dark green (2), black (2), brown (1), and bicolor with a green background and black spot (2) (Figure 1 and Figure 2e, Table S1). Hilum colors ranged from light brown (12) to brown (8), dark brown (2), and black (8) (Figure 2f). Cotyledon color was predominantly yellow among the 30 soybean accessions, with only one accession (‘Qingliang Xifengdou’) displaying green cotyledons (Figure 2g). Dry seeds exhibited diverse shapes, including round, ellipsoid, kidney-shaped, flat round, and flat ellipsoid. Among these, ellipsoid (11 accessions) and round (10 accessions) were the most prevalent (Figure 2h).

Agronomic and yield-related traits also showed substantial variation (Table 1 and Table S2). The growth period ranged from 99.33 to 134.00 days, with a mean of 118.01 days and a coefficient of variation (CV) of 6.27% (Table 1), indicating that most accessions exhibited a long growth duration, making them suitable for summer cropping. Plant height varied considerably, from 47.77 cm to 110.50 cm, with a mean of 76.01 cm and a CV of 23.36% (Table 1). All accessions displayed thick stems, averaging 13.24 mm in diameter, with relatively low variation (CV = 11.73%) (Table 1). The number of nodes on main stem showed noticeable differences, ranging between 12.33 and 22.67, with a mean of 17.29 and a CV of 15.59% (Table 1). The branch number varied from 2.67 to 7.33, averaging 4.26 and showing high variability (CV = 25.83%) (Table 1). The first pod height ranged from 5.77 cm to 18.36 cm and exhibited the highest variability among traits (CV = 31.43%) (Table 1).

Notable differences were recorded for yield components (Table S2 and Table 1). The number of effective pods per plant varied widely from 46.07 to 132.37, with a mean of 77.75 and a CV of 31.44% (Table 1), reflecting significant differences in pod-setting ability among 30 soybean accessions. Seven accessions, including ‘Bianqing Maodou’, ‘Lvxiang Susudou’, ‘Hei Maodou’, ‘Jinhui Maodou’, ‘Qingsu 7’, ‘Changshengguo Maodou’, and ‘Heidou’, produced more than 100 pods per plant (Table S2). Pod size was generally large across the 30 soybean resources, with pod length ranging from 5.60 cm to 7.80 cm and pod width from 1.37 cm to 1.77 cm. The low CV values for pod length (7.67%) and pod width (7.04%) suggest limited variation in these traits (Table 1). In addition, the average 100-grain weight was 86.93 g for fresh seeds and 41.83 g for dry seeds, with coefficients of variation (CVs) of 15.05% and 12.50%, respectively (Table 1). Among them, ‘Dashu Maodou’, ‘Baimang Liuyuebai’, and ‘Caojing Huadou’ had the largest seeds, with fresh 100-seed weights exceeding 100 g and dry 100-seed weights around 50 g (Table S2). This combination of appreciable variation and superior-performing accessions highlights promising potential for selective genetic improvement in seed size.

**Figure 3:**
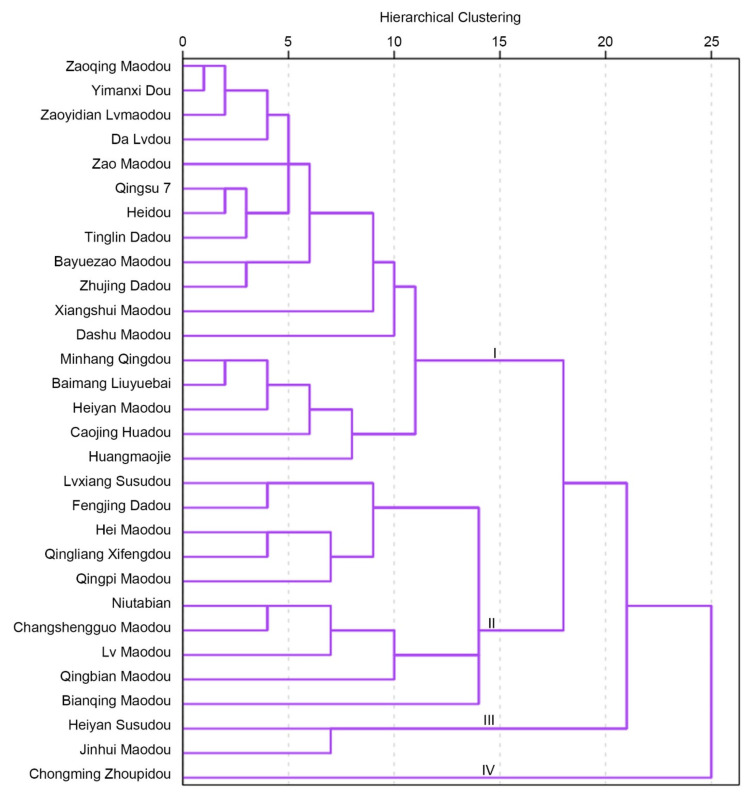
Hierarchical clustering dendrogram of 30 vegetable soybean landraces based on main agronomic and yield traits.

Based on the main agronomic and yield traits, the 30 vegetable soybean accessions were clustered into four main groups at a distance threshold of 15 (Figure 3). Group 1 mainly comprised 17 accessions (‘Zaoqing Maodou’, ‘Yimanxi Dou’, ‘Zaoyidian Lvmaodou’, etc.); Group 2 was made up of 10 accessions (‘Lvxiang Susudou’, ‘Fengjing Dadou’, ‘Hei Maodou’, etc.); and Group 3 contained only two accessions (‘Heiyan Susudou’ and ‘Jinhui Maodou’), which exhibited the highest main stem node number and first pod height, along with distinct compositional characteristics that clearly distinguished this group from the others. Group 4 was represented by a single, distinct accession, ‘Chongming Zhoupidou’, which displayed the shortest growth period and the smallest stem diameter, standard pod length, and standard pod width.

### 3.2. Principal Component Analysis of the Main Agronomic and Yield Traits

Principal component analysis (PCA) was performed to reduce the dimensionality of 11 agronomic and yield traits, and the first four principal components (PCs) collectively explained 79.61% of the total variance (Table 2, Figure S1). PC1 explained 34.08% of the total variance, with strong positive loadings on plant height (0.85), number of nodes on the main stem (0.79), and number of effective pods per plant (0.81), traits reflecting vegetative growth vigor and early reproductive potential. PC2 explained 20.37% of the total variance, dominated by positive loadings on length of standard pod (0.82) and width of standard pod (0.87), and thereby representing pod size characteristics. PC3 explained 15.93% of the variance, with high positive loadings on stem diameter (0.68) and the height of podding (0.70), alongside a positive loading on 100-grain weight of dry grain (0.60). As such, PC3 was characterized as a ‘stem-podding architecture and grain weight’ axis. PC4 (9.22% variance) had moderate positive loadings on the number of branches (0.43) and 100-grain weight of fresh grain (0.49), representing a secondary axis of branching and fresh grain weight.

**Figure 4:**
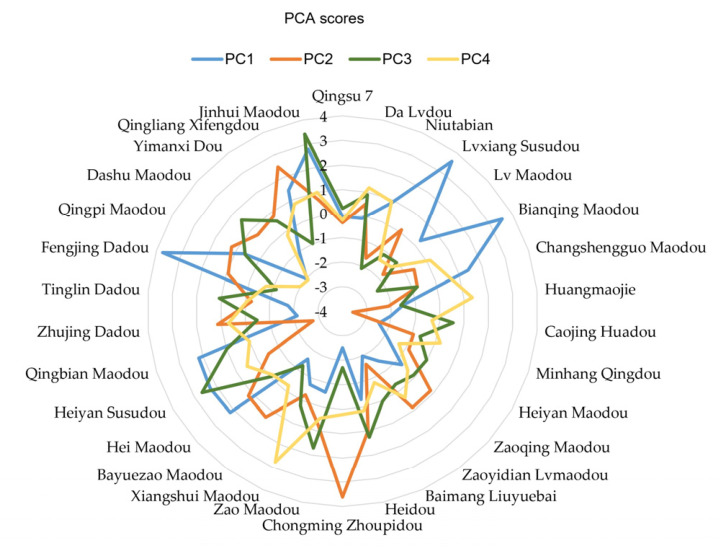
Radar plot of PCA scores for all vegetable soybean accessions along PC1–PC4, which together accounted for 79.61% of the total variance. Blue, orange, green and yellow lines denote the PC1, PC2, PC3, and PC4 scores, respectively.

The radial PCA score plot (Figure 4, Table S3) visualized the differentiation pattern of all vegetable soybean accessions along the four principal component axes, with each colored polygon corresponding to PC1 (blue), PC2 (orange), PC3 (green), and PC4 (yellow). Distinct divergent profiles were observed among germplasm entries. Accessions such as ‘Changshengguo Maodou’, ‘Fengjing Dadou’, and ‘Tinglin Dadou’ displayed far-extended blue outlines, indicating superior performance in PC1-related plant growth and pod yield traits. Notably, the accession ‘Chongming Zhoupidou’ showed prominent orange polygon expansion, which represented larger pod size reflected by high PC2 scores. Although its standard pod length and width were the lowest across all accessions (pod size is a strong positive contributor to PC2), its high fresh and dry 100-grain weights, moderate stem diameter and low first pod height jointly generated a strong positive comprehensive effect that offset the negative contribution of its tiny pods, resulting in an outstanding PC2 value. Accessions such as ‘Heiyan Susudou and ‘Qingsu 7’ showed stretched green edges, demonstrating stronger stem development and heavier dry grain weight associated with PC3. Meanwhile, accessions such as ‘Xiangshui Maodou’ exhibited expanded yellow rays and high PC4 scores. They were inferior in branching number and fresh 100-grain weight (positive traits for PC4), but their low first pod height and small pod size greatly increased PC4 values. Overall, high scores in individual PCs reflected integrated performance across various measured traits rather than superior performance in a single targeted trait.

### 3.3. Correlation Analysis of the Main Agronomic and Yield Traits

Pearson correlation analysis identified several significant relationships among the agronomic and yield-related traits examined (Table 3). Growth period exhibited a highly significant positive correlation with plant height (*r* = 0.56, *p* < 0.01) and length of standard pod (*r* = 0.51, *p* < 0.01), while showing a moderate positive correlation with the number of effective pods per plant (*r* = 0.46, *p* < 0.05). Plant height was strongly positively correlated with the number of nodes on the main stem (*r* = 0.69, *p* < 0.01) and number of branches (*r* = 0.60, *p* < 0.01), and also significantly correlated with the number of effective pods per plant (*r* = 0.70, *p* < 0.01). Furthermore, it showed a highly significant positive correlation with the first pod height (*r* = 0.52, *p* < 0.01) and length of standard pod (*r* = 0.49, *p* < 0.01). In addition, the number of nodes on the main stem was significantly positively correlated with the first pod height (*r* = 0.46, *p* < 0.05) and the number of effective pods per plant (*r* = 0.54), and negatively correlated with SFW (*r* = −0.36, *p* < 0.05). Moreover, the strongest correlation was observed between the length and width of the standard pod (*r* = 0.66, *p* < 0.01), reflecting coordinated development of pod size traits. SFW and SDW also presented an extremely strong positive correlation (*r* = 0.68, *p* < 0.01), indicating high consistency between fresh and dry grain weight. Most yield-related traits (e.g., number of effective pods per plant) were positively correlated with vegetative growth traits (e.g., plant height, node number), while grain weight traits showed weak negative correlations with some pod/plant traits (e.g., number of effective pods per plant), suggesting a potential trade-off between pod number and grain weight.

### 3.4. Taste Quality and Nutritional Profiles of Fresh Vegetable Soybean Seeds

**Figure 5:**
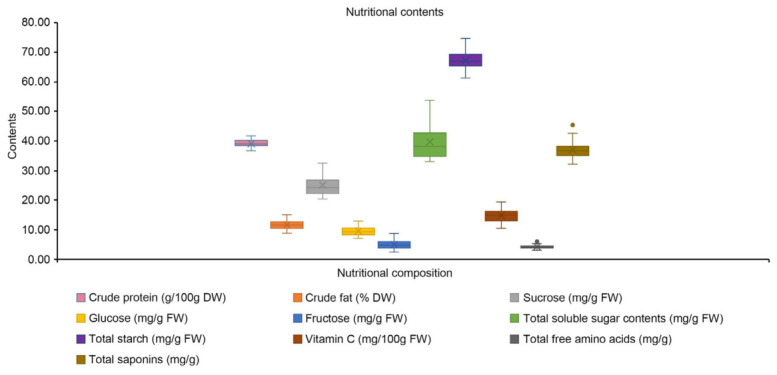
The average concentrations (mean values) of ten major nutritional compounds. Values are presented in their respective units as indicated. Error bars represent the standard deviation from the mean, indicating the variability among different accessions.

The taste-related and nutritional attributes of fresh seeds from 30 soybean germplasms were quantified (Table 4 and Table S4, Figure 5). Crude protein showed extremely limited phenotypic variation across all tested accessions, with highly consistent protein deposition among genotypes. The low coefficient of variation (CV = 3.24%) verified vegetable soybean as an outstanding high-protein dietary raw material (Table 4 and Table S4). By contrast, crude fat exhibited pronounced variation, with the ‘Qingpi Maodou’ identified as having the highest crude fat content (15.08 g/100 g DW) (Table 4 and Table S4).

The soluble sugar profile of vegetable soybean included fructose, glucose, and sucrose, of which sucrose dominated the soluble sugar pool in all accessions (Figure S2). Clear disparities were observed in both total soluble sugar yield and individual sugar proportions. ‘Heiyan Susudou’ showed the maximum contents of total soluble sugar, sucrose, and glucose, while ‘Qingsu 7’ contained the highest fructose level at 8.78 mg/100 g FW (Table S4). Total starch was the predominant carbohydrate, and showed moderate genetic stability with narrow inter-accession variation. The two extreme starch phenotypes were separately found in ‘Qingsu 7’ (61.32 mg/g FW) and ‘Zao Maodou’ (74.69 mg/g FW).

Regarding micronutrients and bioactive compounds, vitamin C and total free amino acids both exhibited relatively high inter-genotype variability. Vitamin C content varied widely from 10.51 mg/100 g FW (‘Jingliang Xifengdou’) to 19.33 mg/100 g FW (‘Xiangshui Maodou’) (Figure 4, Table 4). Notably, ‘Xiangshui Maodou’ had the highest concentration of free amino acids (6.00 mg/g), suggesting superior umami potential (Figure 5, Table 4). Total saponins, nutritionally relevant bioactive compounds, displayed weak genotypic differentiation (CV < 10%), with ‘Jongming Zhaopidou’ showing the highest saponin content (Figure 5, Table 4).

**Figure 6:**
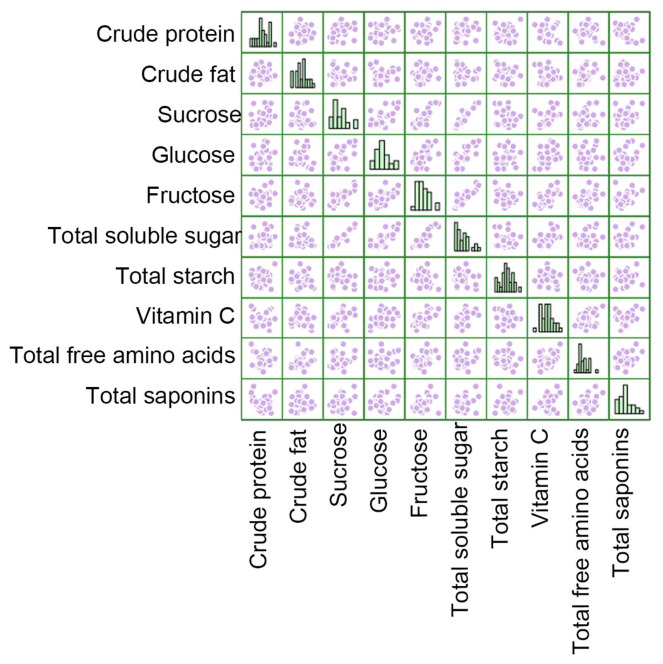
Scatter plot matrix of nutritional components. This matrix plot displays the pairwise relationships between 10 nutritional/bioactive components (rows/columns: crude protein, crude fat, sucrose, glucose, fructose, total soluble sugar, total starch, vitamin C, total free amino acids, total saponins). Each relationship has: (1) a scatter plot (purple points) showing the bivariate distribution of two components; (2) a bar plot (green bars) indicating the univariate distribution (diagonal cells, representing a component’s own distribution). The intensity of point clustering in off-diagonal cells reflects the strength of correlation between two components.

To characterize the nutritional component profiles, we analyzed the levels of 10 key nutrients (including macronutrients, sugars, vitamins, and functional compounds) across accessions, and visualized their distribution patterns via a scatter plot matrix (Figure 6, Table S5). Strong positive associations were observed among soluble sugars: sucrose exhibited highly significant correlations with glucose (*r* = 0.58, *p* < 0.01) and fructose (*r* = 0.75, *p* < 0.01), while the total sugar content was strongly correlated with sucrose (*r* = 0.95, *p* < 0.01), glucose (*r* = 0.77, *p* < 0.01), and fructose (*r* = 0.86, *p* < 0.01). These results align with the compositional contribution of monosaccharides (glucose, fructose) and disaccharides (sucrose) to total sugar content. Vitamin C also showed significant positive correlations with soluble sugars, namely, sucrose (*r* = 0.48, *p* < 0.01), glucose (*r* = 0.43, *p* < 0.05), fructose (*r* = 0.59, *p* < 0.01), and total sugar (*r* = 0.55, *p* < 0.01). For total free amino acids, significant positive correlations were detected with fructose (*r* = 0.42, *p* < 0.05), total sugar (*r* = 0.33, *p* < 0.05), vitamin C (*r* = 0.37, *p* < 0.05), and saponins (*r* = 0.42, *p* < 0.05), suggesting possible metabolic links between nitrogenous compounds, secondary metabolites, and soluble nutrients. In contrast, starch displayed weak or negative correlations with most components (e.g., *r* = −0.19 with fructose; *r* = −0.20 with amino acids), consistent with its role as a storage polysaccharide that may not co-synthesize with soluble nutrients. Protein and fat showed limited correlations with other components (all |r| < 0.31), indicating their independent accumulation patterns among 30 accessions.

### 3.5. Variation in Isoflavone Content and Composition Across Vegetable Soybean Accessions

**Figure 7:**
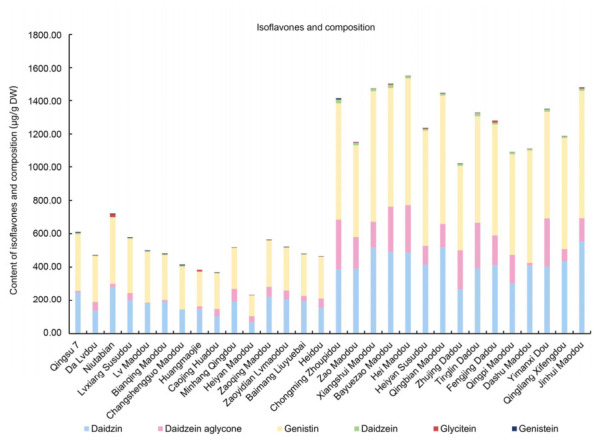
Isoflavone component profiles of 30 vegetable soybean accessions.

Soybean isoflavones (secondary metabolites in soybean) exhibited substantial diversity among the 30 evaluated accessions (Figure 7, Table 5 and Table S6). The evaluated profiles included those of daidzin, daidzein aglycone, genistin, daidzein, glycitein, and genistein. The total isoflavone content ranged widely, from 233.66 to 1550.70 μg/g dry weight (DW), averaging 897.72 μg/g DW with a CV of 49.05%.

Glucoside-type isoflavones predominated among the six endogenous isoflavone derivatives profiled across the 30 soybean accessions. Genistin showed the maximum average content of 471.19 μg/g DW, with its concentration ranging broadly from 127.51 to 782.11 μg/g DW, and thus serves as the primary contributor to total accumulated isoflavones. Daidzin and daidzein aglycone rank as the second and third most abundant isoflavone derivatives, with respective mean concentrations of 301.12 μg/g DW (range: 67.46–555.42 μg/g DW) and 112.13 μg/g DW (range: 1.61–299.77 μg/g DW). These three compounds collectively accounted for more than 98% of the total isoflavones, constituting the major metabolites relevant for functional food applications. In stark contrast, the three free aglycone isoflavones (daidzein, glycitein and genistein) occurred at trace levels throughout all germplasm materials, with average contents only reaching 8.25, 2.79 and 2.23 μg/g DW, respectively. Despite their low absolute concentrations, these minor isoflavone fractions displayed far greater inter-accession fluctuation, as reflected by their elevated coefficients of variation (CVs) of 72.74%, 135.15% and 68.64% (Table 5).

Accession-specific profiles further underscored this heterogeneity (Figure 6, Table S6). Certain accessions, such as ‘Bayuezao Mao dou’ and ‘Hei Mao dou’, accumulated exceptionally high total isoflavone levels (>1500 μg/g DW), driven primarily by elevated genistin and daidzein aglycone. Conversely, accessions like ‘Heiyan Maodou’, ‘Caojing Huadou’ and ‘Huangmaojie’ contained <400 μg/g DW, with reduced accumulation across all components.

Correlation analysis between total isoflavones and core nutritional traits was further performed (Table S7). Total isoflavone content exhibited significant positive correlations with crude fat (*r* = 0.43, *p* < 0.05) and total saponins (*r* = 0.45, *p* < 0.05), and highly significant positive correlations with sucrose (*r* = 0.52, *p* < 0.01), fructose (*r* = 0.55, *p* < 0.01) and total soluble sugar (*r* = 0.56, *p* < 0.01).

## 4. Discussion

### 4.1. Agronomic Performance and Yield Architecture of Shanghai-Native Vegetable Soybean Landraces

Local germplasm resources are vital reservoirs of phenotypic and nutritional diversity, playing a crucial role in crop breeding and agricultural sustainability. This study systematically evaluated 30 soybean accessions (29 Shanghai-native landraces and one control). The wide range observed for key agronomic traits, such as growth period, plant architecture, and yield components, confirms significant phenotypic plasticity among the landraces, which is consistent with the genetic variability reported in previous studies on regional soybean germplasms [6,17]. The clustering analysis revealed four distinct groups, with Group 3 (‘Heiyan Susudou’ and ‘Jinhui Maodou’) and Group 4 (‘Chongming Zhoupidou’) representing unique genetic architectures, potentially harboring novel alleles for targeted trait enhancement.

PCA reduced the dimensionality of 11 agronomic and yield traits into four principal components explaining 79.61% of the total variance, which efficiently captured the key sources of phenotypic variation. PC1, dominated by plant height, the number of nodes on the main stem, and number of effective pods per plant, reflects vegetative growth vigor and early reproductive potential. This is consistent with previous findings that vegetative growth traits are closely linked to yield potential [17]. The strong positive correlations (*r* = 0.54–0.70, *p* < 0.01) observed between plant height, node number, and pod number suggest that vegetative vigor is a key driver of yield potential in these genotypes. Notably, the strong correlation among these three traits reflected by PC1 represents a synergistic outcome of morphological dependency, genetic linkage, and co-developmental regulation operating at multiple levels, rather than being determined by any single mechanism in isolation. At the morphological level, plant height is primarily determined by the main stem node number combined with internode length, while soybean pods are predominantly borne at nodes. Consequently, the main stem node number serves as the morphological foundation determining the potential effective pod number. Extensive statistical analyses, including those conducted as part of the present study, have consistently confirmed highly significant positive correlations between plant height and main stem node number, as well as between main stem node number and effective pod number per plant, reflecting the inherent morphological dependency among these traits [29]. At the genetic level, all three traits are typical quantitative traits controlled by multiple genes/QTLs. Previous studies have demonstrated that QTLs governing different traits frequently exhibit co-localization on chromosomes, i.e., residing in identical or adjacent regions, providing direct genetic evidence for linkage among these traits [30]. Furthermore, pleiotropic effects occur where a single gene influences multiple traits simultaneously. For instance, the *Dt1* gene located at the qHub_5 locus not only regulates plant height and node number but also concurrently affects internode length and pod-bearing range, clearly demonstrating the capacity of one gene to coordinately modulate plant height, node number, and pod distribution and, thus, serving as compelling evidence for co-developmental regulation [31]. In conclusion, the trait associations revealed by PC1 reflect a multi-layered synergistic effect integrating morphological structural constraints, shared genetic elements, and integrated developmental regulatory networks, rather than a simple manifestation of unidirectional causal relationships. PC2, representing pod size (pod length and width), reflects the uniformity of pod traits among Shanghai-native landraces, which is a favorable characteristic for commercial production. PC3 (stem diameter, first pod height, and dry 100-grain weight) and PC4 (branch number and fresh 100-grain weight) further reveal the complexity of trait relationships, providing insights for multi-trait selection in breeding. However, the weak negative correlations between grain weight and some yield-related traits hint at potential trade-offs, which is a critical consideration for breeders aiming to concurrently improve both pod number and seed size. The pronounced variability in first pod height (CV = 31.43%) offers potential for the development of cultivars adapted to different harvesting mechanization systems.

### 4.2. Nutritional Quality and Consumer Acceptability

Beyond agronomic performance, the nutritional profiling revealed marked genotypic differences in components directly governing eating quality and health-promoting value. Humans require large amounts of macronutrients, as they provide energy, maintain fundamental body structure, help prevent diseases and allow for normal functioning of the body. Additionally, micronutrients are primarily obtained from external sources, with the most important source being food [32]. The nutritional quality of vegetable soybean is a key driver of its global demand, and our results highlight the diverse nutritional profiles of Shanghai-native landraces.

Crude protein content was relatively high with low variation (36.78~41.74 g/100 g FW, CV = 3.24%) across landraces, suggesting that vegetable soybean could be considered a valuable and reliable plant-based protein source. This finding is consistent with previous studies on vegetable soybean [32,33]. Comparatively, the protein content of vegetable soybean in our study is much higher when compared to that of other legumes such as common bean (16.70–27.20% DW), bambara groundnut (17.00–17.30% DW), cowpea (20.90–24.70% DW), pea (23.30–26.00% DW), and lentils (25.60–28.90% DW) [34,35], as well as higher than that in staple food and some kinds of meat [36]. Furthermore, vegetable soybean was confirmed to be a consistently high-protein food source, with relatively stable protein levels across different accessions.

Crude fat and crude protein determine the mouthfeel or texture of vegetable soybeans [13]. Our analysis showed that crude fat exhibited higher plasticity (CV = 14.31%), with ‘Qingpi Maodou’ having the highest content (15.08% FW). This substantial variation underscores the potential for breeding cultivars with tailored fat levels to meet different consumer preferences.

Soluble sugars are the main contributors to the sweetness of vegetable soybean [13,15]. Sucrose emerged as the dominant soluble sugar in all landraces, showing a strong correlation with total sugar content, consistent with previous studies [12,13]. ‘Heiyan Susudou’ exhibited a relatively superior sugar profile. Fructose was found to be the least abundant sugar, whereas ‘Qingsu 7’ showed the highest fructose level, which may contribute to its unique flavor.

Total free amino acids, which contribute to umami and flavor complexity [13,15], also showed substantial variation (CV = 15.56%). Notably, the landrace ‘Xiangshui Maodou’ was identified to concurrently accumulate high levels of free amino acids and vitamin C, positioning it as a promising candidate for breeding vegetable soybean with superior flavor and nutritional quality.

Total starch content was relatively stable (CV = 4.80%), consistent with its role as a core storage carbohydrate as detailed in a previous study [35]. Total saponins, bioactive compounds with health benefits, showed moderate variability (CV = 8.33%), with ‘Jongming Zhaopidou’ having the highest content. Vitamin C content varied widely (CV = 14.27%), with ‘Xiangshui Maodou’ having the highest level (19.33 mg/100 g FW). Based on a typical serving size of 100 g fresh vegetable soybean, the accession with the lowest vitamin C content would provide approximately 10.5% of the daily recommended nutrient intake (RNI), whereas the highest-content accession would contribute around 19.3%. This difference corresponds to an additional 8.8 mg of vitamin C per 100 g serving. Although this absolute difference is relatively modest, it may still carry nutritional relevance for consumers aiming to optimize their micronutrient intake through dietary selection, especially among populations with inadequate fruit and vegetable consumption. Vitamin C is an essential antioxidant, and high-vitamin C cultivars have enhanced nutritional value [37]. The significant positive correlation between vitamin C and soluble sugars (*r* = 0.55) suggests coordinated metabolic pathways that could be exploited for simultaneous improvement in nutritional and sensory attributes.

### 4.3. Isoflavone Variation and Functional Food Potential

The substantial diversity in isoflavone profiles observed among Shanghai landraces highlights their significant potential for enabling the development of specialized functional vegetable soybean products. The total isoflavone content varied nearly seven-fold between accessions (234–1551 µg/g DW), with a CV of 49.05%. This magnitude of variation is greater than that reported in previous studies on vegetable and grain soybeans [18,28,38]. In addition, glycosylated forms (genistin, daidzin) constituted over 98% of the composition. Glycosylated isoflavones are generally less bitter than their aglycone counterparts (e.g., daidzein, genistein) and are more stable during fresh consumption and mild processing [39]. Notably, landraces such as ‘Bayuejiao Maodou’ and ‘Hei Maodou’ accumulated over 1500 μg/g DW of total isoflavones, exceeding the levels of most commercial cultivars and positioning them as promising candidates for high-isoflavone functional food development. Soybean isoflavones have been shown to exhibit pharmacological activities, including antioxidant, cancer-preventive, and anticarcinogenic properties [28,40].

## 5. Conclusions

In conclusion, the Shanghai-native vegetable soybean landraces assessed in this study exhibit rich phenotypic and nutritional diversity. The identified trait correlations and trade-offs provide a foundation for the development of high-yield, high-quality, and market-targeted cultivars. Based on the multi-trait evaluation, six priority accessions were identified: ‘Xiangshui Maodou’ (flavor and nutritional quality), ‘Heiyan Susudou’ (sweetness), ‘Hei Maodou’ (dual-purpose: yield and functional food), ‘Dashu Maodou’, ‘Baimang Liuyuebai’, and ‘Caojing Huadou’ (seed size). These research results serve to strengthen the utilization of local germplasm, support sustainable vegetable soybean production, and meet the growing global demand for nutritious, sensory-optimized food crops.

## Figures and Tables

**Table 1:** Comparison of the 30 vegetable soybeans agronomic traits.

Traits	Min	Max	Mean	STDEV	CV (%)
Growth period (d)	99.33	134.00	118.01	7.40	6.27
Plant height (cm)	47.77	110.50	76.01	17.76	23.36
Stem diameter (mm)	10.00	18.00	13.24	1.55	11.73
Number of nodes on main stem	12.33	22.67	17.29	2.69	15.59
Number of branches	2.67	7.33	4.26	1.10	25.83
First pod height (cm)	5.77	18.63	10.81	3.40	31.43
Number of effective pods per plant	46.07	132.37	77.75	24.44	31.44
Length of standard pod (cm)	5.60	7.80	6.50	0.50	7.67
Width of standard pod (cm)	1.37	1.77	1.53	0.11	7.04
100-seed weight of fresh seeds (g)	60.80	110.63	86.93	13.08	15.05
100-seed weight of dry seeds (g)	33.30	54.73	41.83	5.23	12.50

**Table 2:** PCA of main agronomic and yield traits.

Traits	1	2	3	4
Growth period	0.59	0.50	−0.43	0.05
Plant height	0.85	0.06	−0.05	0.35
Stem diameter	0.52	0.30	0.68	−0.18
Number of nodes on main stem	0.79	−0.19	0.22	−0.03
Number of branches	0.67	−0.06	0.01	0.43
Height of podding	0.32	−0.32	0.70	−0.40
Number of effective pods per plant	0.81	0.03	0.00	0.09
Length of standard pod	0.31	0.82	0.03	−0.27
Width of standard pod	−0.13	0.87	−0.15	−0.25
100-grain weight of fresh grain	−0.46	0.45	0.42	0.49
100-grain weight of dry grain	−0.49	0.33	0.60	0.34
Contribution rate (%)	34.08	20.37	15.93	9.22
Cumulative contribution rate (%)	34.08	54.45	70.39	79.61

**Table 3:** Correlation analysis of the main agronomic and yield traits in vegetable soybeans.

Pearson Correlation	Growth Period	Plant Height	Stem Diameter	Number of Nodes on Main Stem	Number of Branches	First Pod Height	Number of Effective Pods per Plant	Length of Standard Pod	Width of Standard Pod	SFW	SDW
Growth period	1	0.56 **	0.12	0.33	0.28	−0.22	0.46 *	0.51 **	0.36 *	−0.17	−0.30
Plant height	0.56 **	1	0.31	0.69 **	0.60 **	0.11	0.70 **	0.17	−0.08	−0.20	−0.29
Stem diameter	0.12	0.31	1	0.42 *	0.32	0.52 **	0.43 *	0.49 **	0.08	0.04	0.17
Number of nodes on main stem	0.33	0.69 **	0.42 *	1	0.40 *	0.46 *	0.54 **	0.04	−0.23	−0.36 *	−0.26
Number of branches	0.28	0.60 **	0.32	0.40 *	1	0.07	0.45 *	0.18	−0.28	−0.19	−0.27
First pod height	−0.22	0.11	0.52 **	0.46 *	0.07	1	0.18	−0.05	−0.29	−0.12	−0.01
Number of effective pods per plant	0.46 *	0.70 **	0.43 *	0.54 **	0.45 *	0.18	1	0.17	−0.07	−0.31	−0.33
Length of standard pod	0.51 **	0.17	0.49 **	0.04	0.18	−0.05	0.17	1	0.66 **	0.10	−0.01
Width of standard pod	0.36 *	−0.08	0.08	−0.23	−0.28	−0.29	−0.07	0.66 **	1	0.26	0.20
SFW	−0.17	−0.20	0.04	−0.36 *	−0.19	−0.12	−0.31	0.10	0.26	1	0.68 **
SDW	−0.30	−0.29	0.17	−0.26	−0.27	−0.01	−0.33	−0.01	0.20	0.68 **	1

**: The correlation is significant at the 0.01 level; *: the correlation is significant at the 0.5 level.

**Table 4:** Variability in nutritional traits among 30 accessions.

Traits	Min	Max	Mean	STDEV	CV (%)
Crude protein (g/100 g DW)	36.78	41.74	39.12	1.27	3.24
Crude fat (g/100 g DW)	8.85	15.08	11.66	1.67	14.31
Sucrose (mg/g FW)	20.40	32.63	25.18	3.40	13.52
Glucose (mg/g FW)	7.07	12.93	9.55	1.57	16.41
Fructose (mg/g FW)	2.50	8.78	5.00	1.48	29.61
Total soluble sugar content (mg/g FW)	33.14	53.61	39.75	5.72	14.38
Total starch (mg/g FW)	61.32	74.69	67.24	3.23	4.80
Vitamin C (mg/100 g FW)	10.51	19.33	14.85	2.12	14.27
Total free amino acids (mg/g)	3.09	6.00	4.27	0.67	15.56
Total saponins (mg/g)	32.23	45.35	37.08	3.09	8.33

**Table 5:** Variation in isoflavone and composition among 30 soybean accessions (μg/g DW).

Traits	Min	Max	Mean	STDEV	CV (%)
Daidzin	67.46	555.42	301.12	143.09	47.52
Daidzein aglycone	1.61	299.77	112.13	99.67	88.89
Genistin	127.51	782.11	471.19	219.02	46.48
Daidzein	0.91	20.99	8.25	6.00	72.74
Glycitein	0.40	17.52	2.79	3.77	135.15
Genistein	0.02	5.86	2.23	1.53	68.64
Total isoflavones	233.66	1550.70	897.72	440.32	49.05

## Data Availability

The original contributions presented in this study are included in the article/Supplementary Material. Further inquiries can be directed to the corresponding author.

## Supplementary Materials

The following supporting information can be downloaded at: https://www.mdpi.com/article/10.3390/foods15132382/s1: Figure S1: PCA plots of 30 soybean accessions based on 11 agronomic and yield-related traits; Figure S2: Soluble sugar components (sucrose, glucose, and fructose) of 30 vegetable soybean accessions; Table S1: Geographical origin and phenotypic traits of the 30 soybean accessions; Table S2: Agronomic and yield-related traits of the 30 soybean accessions; Table S3: Principal component scores of the 30 soybean accessions; Table S4: Taste quality and nutritional contents of the seeds of the 30 soybean accessions; Table S5: Correlation analysis of taste quality and nutritional profiles of fresh vegetable soybean seeds; Table S6: Contents of isoflavones and their composition in 30 soybean accessions; Table S7: Correlation analysis of the total isoflavone content and major nutritional quality traits in soybeans.

## Abbreviations

The following abbreviations are used in this manuscript:

PCA	principal component analysis
CV	coefficient of variation
PCs	principal components
SFW	100-seed fresh weight
SDW	100-seed dry weight

## References

[1] Hijrah M., Wirnas D., Trikoesoemaningtyas, Sopandie D. (2024). Diversity of morphological, agronomic, and quality traits of soybean (*Glycine max* L.) and their potential as edamame. SABRAO J. Breed. Genet..

[2] Liu N., Feng W., Zhang G., Gao P., Lian L., Yuan J., Liu X., Ni X., Wang H., Ou J. (2025). Genomic and Population Evidence Uncovers Divergent Improvement of Vegetable Soybean from Grain Soybean. Mol. Plant.

[3] Chen Z., Zhong W., Zhou Y., Ji P., Wan Y., Shi S., Yang Z., Gong Y., Mu F., Chen S. (2022). Integrative Analysis of metabolome and transcriptome reveals the improvements of seed quality in vegetable soybean (*Glycine max* (L.) Merr.). Phytochemistry.

[4] Williams M.M. (2015). Phenomorphological Characterization of Vegetable Soybean Germplasm Lines for Commercial Production. Crop Sci..

[5] Miles C.A., O’Dea J., Daniels C.H., King J. (2018). Edamame; A pacific northwest extension publication.

[6] Zeipiņa S., Alsiņa I., Lepse L. (2017). Insight in edamame yield and quality parameters: A review. Agric. Sci..

[7] Carneiro R.C., Duncan S.E., O’Keefe S.F., Yin Y., Neill C.L., Zhang B. (2020). Sensory and Consumer Studies in Plant Breeding: A Guidance for Edamame Development in the U.S. Front. Sustain. Food Syst..

[8] Gondim-Tomaz R.M.A., Braga N.R., Carvalho C.R.L., Gallo P.B., Erismann N.d.M. (2022). Phytochemical evaluation of lipoxygenase-free soybean genotypes for human consumption. Braz. J. Food Technol..

[9] Xu Y., Cartier A., Kibet D., Jordan K., Hakala I., Davis S., Sismour E., Kering M., Rutto L. (2016). Physical and nutritional properties of edamame seeds as influenced by stage of development. J. Food Meas. Charact..

[10] Li Y.-S., Du M., Zhang Q.-Y., Wang G.-H., Hashemi M., Liu X.-B. (2012). Greater differences exist in seed protein, oil, total soluble sugar and sucrose content of vegetable soybean genotypes [*Glycine max* (L.) Merrill] in northeast China. Aust. J. Crop Sci..

[11] Czaikoski K., Leite R.S., Mandarino J.M.G., Carrão-Panizzi M.C., da Silva J.B., Ida E.I. (2013). Canning of vegetable-type soybean in acidified brine: Effect of the addition of sucrose and pasteurisation time on color and other characteristics. Ind. Crop. Prod..

[12] Nair R.M., Boddepalli V.N., Yan M.-R., Kumar V., Gill B., Pan R.S., Wang C., Hartman G.L., e Souza R.S., Somta P. (2023). Global status of vegetable soybean. Plants.

[13] Song J., Liu C., Li D., Gu Z. (2013). Evaluation of sugar, free amino acid, and organic acid compositions of different varieties of vegetable soybean (*Glycine max* [L.] Merr). Ind. Crop. Prod..

[14] Guo J., Rahman A., Mulvaney M.J., Hossain M.M., Basso K., Fethiere R., Babar A. (2020). Evaluation of edamame genotypes suitable for growing in Florida. Agron. J..

[15] Flores D., Giovanni M., Kirk L., Liles G. (2019). Capturing and explaining sensory differences among organically grown vegetable-soybean varieties grown in Northern California. J. Food Sci..

[16] Yu O., McGonigle B. (2005). Metabolic engineering of isoflavone biosynthesis. Adv. Agron..

[17] Li X., Liu K., Rideout S., Rosso L., Zhang B., Welbaum G.E. (2024). Seed physiological traits and environmental factors influence seedling establishment of vegetable soybean (*Glycine max* L.). Front. Plant Sci..

[18] Devi J., Prasad I., Rai N., Singh K.P., Singh N.K., Aravind T. (2025). Vegetable soybean: Priority traits, improvement strategies, and future prospects. Soybean Production Technology.

[19] Ma K., Wang Q.Z., Yang H.J., Gu W., Yu D. (2006). Breeding of a new vegetable soybean variety ‘Qingsu4’ of earliness and good quality and disease resistance. Acta Agric. Shanghai.

[20] Liu C., Zhang Q., Hu Y., Li Y., Liu X. (2025). Sucrose as a key nutritional marker distinguishing vegetable and grain soybeans, regulated by *GmZF-HD1* via *GmSPS17* in seeds. Hortic. Res..

[21] Gao H., Wu G., Wu F., Zhou X., Zhou Y., Xu K., Li Y., Zhang W., Zhao K., Jing Y. (2024). Genome-wide association analysis of yield-related traits and candidate genes in vegetable soybean. Plants.

[22] Li X., Zhou Y., Bu Y., Wang X., Zhang Y., Guo N., Zhao J., Xing H. (2021). Genome-wide association analysis for yield-related traits at the R6 stage in a Chinese soybean mini core collection. Genes Genom..

[23] Jiang G.-L., Rutto L.K., Ren S., Bowen R.A., Berry H., Epps K. (2018). Genetic analysis of edamame seed composition and trait relationships in soybean lines. Euphytica.

[24] Friedman M. (2004). Applications of the ninhydrin reaction for analysis of amino acids, peptides, and proteins to agricultural and biomedical sciences. J. Agric. Food Chem..

[25] Shilpashree N., Devi S.N., Manjunathagowda D.C., Muddappa A., Abdelmohsen S.A.M., Tamam N., Elansary H.O., El-Abedin T.K.Z., Abdelbacki A.M.M., Janhavi V. (2021). Morphological characterization, variability and diversity among vegetable soybean (*Glycine max* L.) genotypes. Plants.

[26] Berhow M.A., Wagner E.D., Vaughn S.F., Plewa M.J. (2000). Characterization and antimutagenic activity of soybean saponins. Mutat. Res. Fundam. Mol. Mech. Mutagen..

[27] Hoeck J.A., Fehr W.R., Murphy P.A., Welke G.A. (2000). Influence of genotype and environment on isoflavone contents of soybean. Crop. Sci..

[28] Kim J.A., Chung I.M. (2007). Change in isoflavone concentration of soybean (*Glycine max* L.) seeds at different growth stages. J. Sci. Food Agric..

[29] Yadav S., Dhall R.K., Singh H., Kumar P., Sharma P., Kumar P., Kumari P., Rana N. (2025). Comprehensive genetic analysis of edible-podded pea genotypes: Variability, heritability, and multivariate approach across two agro-climatic zones in India. Horticulturae.

[30] Ning H., Yuan J., Dong Q., Li W., Xue H., Wang Y., Tian Y., Li W.-X. (2018). Identification of QTLs related to the vertical distribution and seed-set of pod number in soybean [*Glycine max* (L.) Merri]. PLoS ONE.

[31] Niu M., Tian K., Chen Q., Yang C., Zhang M., Sun S., Wang X. (2023). A multi-trait GWAS-based genetic association network controlling soybean architecture and seed traits. Front. Plant Sci..

[32] Agyenim-Boateng K.G., Zhang S., Zhang S., Khattak A.N., Shaibu A., Abdelghany A.M., Qi J., Azam M., Ma C., Feng Y. (2023). The nutritional composition of the vegetable soybean (Maodou) and its Potential in combatting malnutrition. Front. Nutr..

[33] Yu D., Lord N., Polk J., Dhakal K., Li S., Yin Y., Duncan S.E., Wang H., Zhang B., Huang H. (2022). Physical and chemical properties of edamame during bean development and application of spectroscopy-based machine learning methods to predict optimal harvest time. Food Chem..

[34] Baptista A., Pinho O., Pinto E., Casal S., Mota C., Ferreira I.M.P.L.V.O. (2017). Characterization of protein and fat composition of seeds from common beans (*Phaseolus vulgaris* L.), cowpea (*Vigna unguiculata* L. Walp) and bambara groundnuts (*Vigna subterranea* L. Verdc) from Mozambique. J. Food Meas. Charact..

[35] Keskin S.O., Ali T.M., Ahmed J., Shaikh M., Siddiq M., Uebersax M.A. (2021). Physico-chemical and functional properties of legume protein, starch, and dietary fiber—A review. Legume Sci..

[36] Mohanty B.P., Mahanty A., Ganguly S., Mitra T., Karunakaran D., Anandan R. (2019). Nutritional composition of food fishes and their importance in providing food and nutritional security. Food Chem..

[37] Kumar V., Rani A., Dixit A.K., Pratap D., Bhatnagar D. (2010). A Comparative assessment of total phenolic content, ferric reducing-anti-oxidative power, free radical-scavenging activity, vitamin C and isoflavones content in soybean with varying seed coat colour. Food Res. Int..

[38] Kumar V., Rani A., Goyal L., Pratap D., Billore S., Chauhan G. (2011). Evaluation of vegetable-type soybean for sucrose, taste-related amino acids, and isoflavones contents. Int. J. Food Prop..

[39] Dhaubhadel S., McGarvey B.D., Williams R., Gijzen M. (2003). Isoflavonoid biosynthesis and accumulation in developing soybean seeds. Plant Mol. Biol..

[40] Wu H., Zhang Z., Huang H., Li Z. (2017). Health benefits of soy and soy phytochemicals. AME Med. J..

