# Supplementary figures and images for: Comprehensive Evaluation of Agronomic Traits and Nutritional Composition in Summer-Sown Vegetable Soybean Varieties from Shanghai, China
Source: Foods. 2026 Jul 3;15(13):2382. doi: 10.3390/foods15132382 (PMC13362312; doi:10.3390/foods15132382)

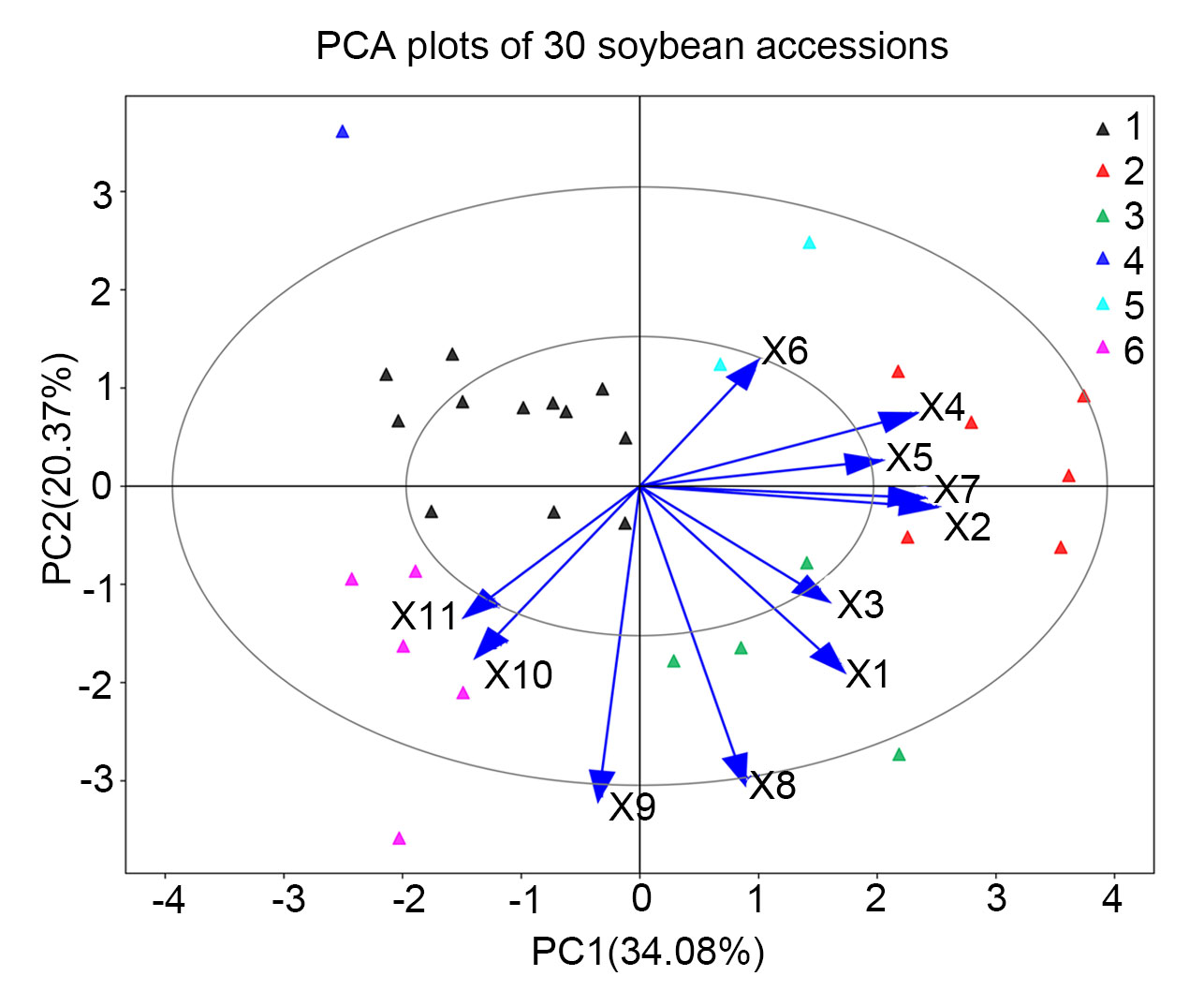

Supplement: Supplementary file 1 [file foods-15-02382-s001.zip › Figure S1-PCA plots of 30 soybean accessions based on 11 agronomic and yield-related traits.jpg]

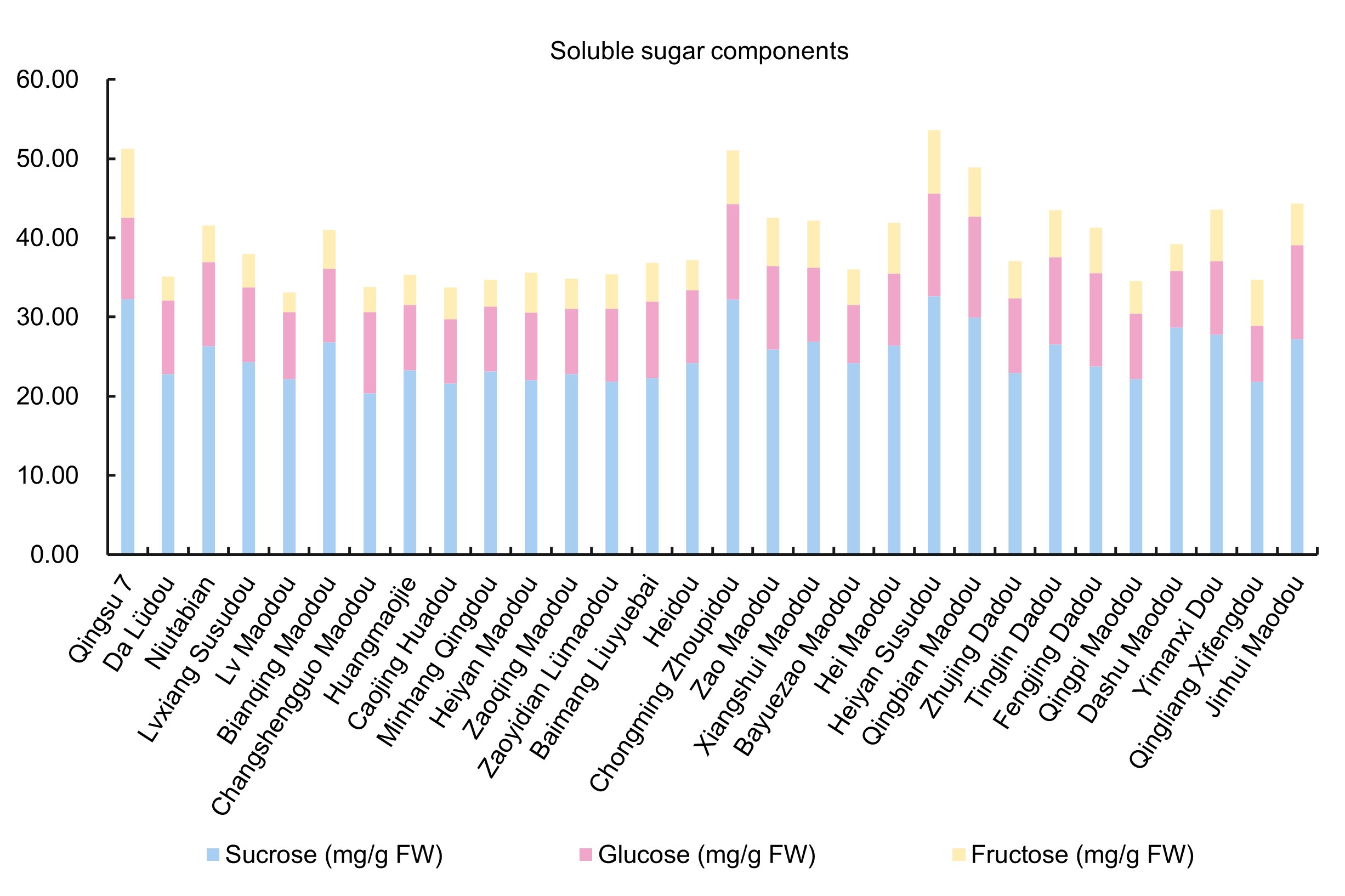

Supplement: Supplementary file 1 [file foods-15-02382-s001.zip › Figure S2-Soluble sugar components (sucrose, glucose, and fructose) of 30 vegetable soybean accessions.jpg]
